# Disability-inclusive graduation programme intervention on social participation among ultra-poor people with disability in North Uganda: a cluster randomized trial

**DOI:** 10.1186/s12916-025-04100-3

**Published:** 2025-04-30

**Authors:** Shanquan Chen, Lena Morgon Banks, Mark T. Carew, Elijah Kipchumba, Calum Davey, Munshi Sulaiman, Hannah Kuper

**Affiliations:** 1 International Centre for Evidence in Disability, London School of Hygiene & Tropical Medicine, London, WC1E 7HT UK; 2 Trinity College Dublin, Dublin, Ireland; 3 National Institute of Teaching, London, UK; 4 Brac Institute of Governance and Development (BIGD), BRAC University, Dhaka, Bangladesh; 5 Independent Evaluation and Research Cell, BRAC Uganda, Kampala, Uganda

**Keywords:** Disability inclusion, Social participation, Cluster randomized trial, Uganda, Low-income countries

## Abstract

**Background:**

People with disabilities encounter significant barriers to social participation due to inaccessible environments and negative attitudes. This study evaluated the effectiveness of a comprehensive disability-inclusive graduation (DIG) programme in enhancing social participation among ultra-poor people with disabilities in rural Uganda.

**Methods:**

A two-arm, cluster-randomized controlled trial was conducted in four Northern Ugandan districts, involving 96 intervention and 89 control clusters. The DIG intervention encompassed four pillars: Livelihoods, Social Protection, Financial Inclusion, and Social Empowerment. The study identified households with disabilities using the Washington Group Short Set questions, verified by BRAC programme managers, comprising 370 working-age people with disabilities in the intervention group and 321 in the control group at baseline. Treatment clusters received an 18-month DIG intervention from December 2020 to June 2022. Social participation was measured using the SINTEF Participation Question Set at baseline, immediately post-intervention, and 16 months post-intervention, covering household and societal participation domains. Intervention effects were analyzed through linear mixed-effects regression models, reporting minimally adjusted and fully adjusted mean differences (MAMD and FAMD) with 95% confidence intervals.

**Results:**

Immediately after the intervention, the DIG programme showed a positive trend in overall social participation (3.04 point increase in intervention group vs. − 0.29 in control), though not statistically significant in fully adjusted analysis (FAMD = 3.14, 95% CI = (− 1.26, 7.54); *p* = 0.17), possibly due to sample size limitations and variability in individual responses. A larger improvement in societal participation was observed favouring the intervention group (5.92 point increase versus 0.21 in control), with the fully adjusted analysis approaching statistical significance (FAMD = 5.84, 95% CI = (− 0.01, 11.69); *p* = 0.05). No significant differences were found in the domain of household participation. At 16 months post-intervention, no significant differences were observed between the intervention and control groups in overall scores or any subdomain, suggesting challenges in maintaining initial improvements over time.

**Conclusions:**

The DIG programme showed short-term positive effects on social participation among ultra-poor people with disabilities, especially in societal engagement. The absence of long-term sustained improvements underscores the complexity of disability inclusion in resource-constrained settings. Future interventions should develop strategies like extended support or booster sessions to maintain initial gains.

**Trial registration:**

Registry for International Development Impact Evaluations (RIDIE-STUDY-ID-626008898983a) and ISRCTN (ISRCTN-78592382).

**Supplementary Information:**

The online version contains supplementary material available at 10.1186/s12916-025-04100-3.

## Background

The United Nations Convention on the Rights of Persons with Disabilities (UNCRPD) defines persons with disabilities as “those who have long-term physical, mental, intellectual or sensory impairments which in interaction with various barriers may hinder their full and effective participation in society on an equal basis with others” [1]. According to the World Health Organization (WHO), approximately 16% of the world’s population, or around 1.3 billion people, live with some form of disability [2]. This prevalence is not uniformly distributed, with an estimated 80% of people with disabilities residing in low- and middle-income countries (LMICs) [2].

Participation is a broad term, and one often difficult to define and measure. It is defined in the WHO’s International Classification of Functioning, Disability and Health (ICF) as “involvement in a life situation” [3]. However, the broad ICF definition encompasses both social participation (e.g. interpersonal and community interactions) as well as non-social forms of participation as activity (e.g. bathing). Social participation is considered an important modifiable determinant of health and wellbeing [4], yet here too there is no universally agreed consensus on definition or conceptualizations, despite its extensive use in the literature [5]. One review covering 43 frameworks of social participation identified its core definitional element as a focus on “the person’s involvement in activities that provide interaction with others in society or the community” [6]. This scope includes forms of community participation that are directed toward improving the conditions of the community and shaping members’ future (e.g., voting) [7]. Social participation also encompasses participation that takes place with family and household members (e.g., taking part in household decision making) [8].

Despite global efforts to promote inclusion, people with disabilities continue to face substantial barriers in social participation. For example, a comparative survey of households with and without disabled members in Liberia showed that disabled respondents felt less included in the community, were less likely to engage in community participation, and felt less included in community decision making compared to non-disabled respondents [9]. The study also identified disparities on dimensions of community participation between disabled respondents and non-disabled members of the same household. Similarly, a study in rural Uganda found that fewer people with disabilities reported being regularly involved in household financial decisions compared to non-disabled individuals [10].

Ultra-poor people with disabilities face a double burden of marginalization through both poverty and disability. They experience greater barriers to social participation due to limited access to assistive devices, transportation challenges, and fewer economic resources to facilitate community engagement [2, 9–11]. Our focus on this specific subgroup addresses the critical intersection of disability and extreme poverty, which remains under-researched despite representing some of the most vulnerable populations in LMICs.

Improving the social participation of people with disabilities is critical. First and foremost, people with disabilities have the right to participate in society on an equal basis as others: the UNCRPD explicitly recognizes the right of people with disabilities to full and effective participation in society. This includes specifically the right to be included in the community. Similarly, the 2030 Agenda for Sustainable Development (SDG) pledges to “leave no one behind”, explicitly including people with disabilities in its vision for inclusive development. Despite the clear importance of promoting social participation of people with disabilities, including to maximize health and well-being, significant challenges remain in developing and implementing effective interventions, particularly in LMICs. In particular, there is a significant lack of rigorous evidence on the effectiveness of interventions to improve social participation of people with disabilities.

A systematic review by Saran et al. (2023) identified 37 studies that evaluated the impact of interventions on improving social inclusion for people with disabilities, but most of these targeted improving specific social or communication skills of people with disabilities, rather than tackling systemic drivers [11]. Furthermore, the majority had a high risk of bias, which collectively underscores the urgent need for more robust research in this area [11]. Recognizing these limitations, some targeted interventions have been developed. Community-based rehabilitation (CBR) programmes, for instance, aim to enhance the participation of people with disabilities through a multifaceted approach, with two meta-analyses suggesting that CBR can positively impact social participation of people with disabilities [12, 13]. Concurrently, disability-inclusive livelihood programmes have emerged, such as Humanity and Inclusion (HI)’s project across five African countries that combined vocational training with advocacy for inclusive employment policies, resulting in improved economic outcomes for people with disabilities [14]. While these represent important advances, research has tended to focus either narrowly on social skills development or primarily on economic outcomes, with fewer studies examining whole approaches that address the interconnected nature of social and economic participation.

The relationship between economic empowerment and social participation is bidirectional. Economic resources can facilitate social participation through means for transportation and community activities, while increased social participation can enhance economic opportunities through expanded networks. However, economic interventions alone often fail to address attitudinal barriers or accessibility challenges, highlighting the need for integrated approaches, particularly for ultra-poor people with disabilities.

This limited evidence base hampers the development and implementation of effective, evidence-based interventions that recognize and address the multifaceted needs of people with disabilities. The current research landscape thus highlights the pressing need for innovative, comprehensive interventions that simultaneously address multiple dimensions of participation for people with disabilities in resource-constrained settings.

Graduation programmes have emerged as a promising approach to address extreme poverty through time-bound, multi-faceted interventions that combine social protection, livelihood development, financial inclusion, and social empowerment components [15]. These programmes aim to “graduate” participants from extreme poverty to sustainable livelihoods through a comprehensive package of support [15]. Disability-inclusive graduation (DIG) programmes specifically adapt this approach to address the unique barriers faced by people with disabilities living in extreme poverty.

This study aims to address these gaps by evaluating the effectiveness of a DIG programme in enhancing the social participation of ultra-poor people with disabilities in Uganda. It assesses changes in areas such as household and societal participation; this study seeks to provide rigorous evidence on the impact of a tailored, disability-inclusive approach to social inclusion. The findings have the potential to inform policy and practice in disability-inclusive development, both in Uganda and in other LMICs, contributing to the broader goal of creating more equitable and inclusive societies for all. Our findings could inform Uganda's implementation of the 2019 Persons with Disabilities Act while influencing international development agencies in designing future disability-inclusive programmes. The evidence may shape disability inclusion frameworks used by organizations like the World Bank and UN agencies, contributing to global advocacy efforts by demonstrating practical approaches to realizing social participation rights in the UNCRPD. Based on the DIG programme’s theory of change, we hypothesized that the intervention would enhance social participation, specifically leading to better social integration in households and communities through its comprehensive support package.

## Methods

### Study design and participants

We conducted a two-arm, parallel cluster-randomized controlled trial in four districts of Northern Uganda: Kiryandongo, Gulu, Nwoya, and Oyam, with an estimated total population of 1.4 million people. The study protocol received ethical approval from the Mildmay Uganda Research Ethics Committee (Reference: 0604–2020), the London School of Hygiene and Tropical Medicine Research Ethics Committee (References: 22,619/RR/21198 and 28,134), and a research permit from the Uganda National Council for Science and Technology (UNCST) (Reference: SS529ES). The study protocol has been previously published [16].

Cluster randomization was employed due to the nature of the DIG programme, which includes components delivered at the village level. Clusters were defined as villages containing 10–75 eligible households, sized to ensure viable operation of village-level interventions while remaining manageable for implementation. When necessary, villages were merged or divided using k-means clustering based on GPS coordinates to create appropriate cluster sizes.

Eligible households met at least three of five criteria: (1) having a person with a disability, (2) being a female-headed household or dependent on earnings from a female member, (3) having children who are out of school, (4) poor housing conditions, and (5) low productive asset endowment. In our DIG programme, ultra-poverty refers to systematic and structural poverty aligned with the notion of poverty traps, rather than stochastic poverty resulting from temporary shocks or seasonal variations. While international standards define extreme poverty as living below USD 1.90 per day, consultation with local authorities indicated this threshold alone might not fully reflect the contextual realities in Uganda. Therefore, ultra-poverty was operationalized through proxy means testing using the five eligibility criteria described above. Our results in Table 1 confirm the compatibility of this proxy approach with international standards, as all selected participants fell below the USD 1.90 per day threshold for extreme poverty.

**Table 1:** Baseline characteristics of index people with disabilities in the DIG intervention and control groups. Data was reported as the mean (standard deviation) or number (percentage)

	**DIG intervention group** **(*n* = 370)**	**Control group** **(*n* = 321)**
**Factors for index people with disabilities**		
**Age (years)**	35.71 (12.42)	34.31 (11.97)
**Sex (= female)**	198 (53.5%)	172 (53.6%)
**Level of education**		
No education	82 (22.2%)	77 (24.0%)
Primary education	226 (61.1%)	208 (64.8%)
Secondary education	56 (15.1%)	31 (9.7%)
Specialized training/bachelor or above	6 (1.6%)	5 (1.6%)
**Marital status**		
Never married	123 (33.2%)	110 (34.3%)
Married/cohabiting	156 (42.2%)	152 (47.4%)
Divorced/separated/widow	91 (24.6%)	59 (18.4%)
**Is household head (= yes)**	173 (46.8%)	141 (43.9%)
**Is project participant (= yes)**	176 (47.6%)	153 (47.7%)
**Household-level factors**		
**Highest level of education**		
No education	3 (0.8%)	5 (1.6%)
Primary education	206 (55.7%)	188 (58.6%)
Secondary education	140 (37.8%)	110 (34.3%)
Specialized training/bachelor or above	21 (5.7%)	18 (5.6%)
**Lives in poverty (= yes)**	370 (100.0%)	321 (100.0%)
**Number of families**	5.78 (2.18)	5.79 (2.38)
Number of children	0.67 (0.91)	0.75 (1.00)
**Per capital income (dollars) per month**	69.33 (80.11)	66.58 (75.16)
**Outcomes**		
**Social participation**	72.45 (25.88)	71.97 (24.94)
Domain 1: Household participation	86.31 (25.27)	85.57 (25.13)
Domain 2: Societal participation	62.06 (33.21)	61.77 (30.86)

Disability was ascertained through a two-part process using the Washington Group Short Set questions [17]. Individuals who reported experiencing “a lot of difficulty” or “cannot do” in at least one of six domains (seeing, hearing, walking, cognition, self-care, and communication) were screened as positive for disability [17], with subsequent verification by a BRAC programme manager.

Villages were merged or divided into clusters using k-means clustering based on GPS coordinates of eligible households. From 156 villages, 185 artificial clusters were created. A single individual within each household was designated as the “project participant” and primary recipient of training and enterprise support, with women and people with disabilities prioritized. In cases where a person with disability was unable to manage available enterprise options, their primary caregiver was suggested as the participant instead. Children (below 18 years) were not eligible to be project participants.

### Randomization and masking

**Fig. 1:**
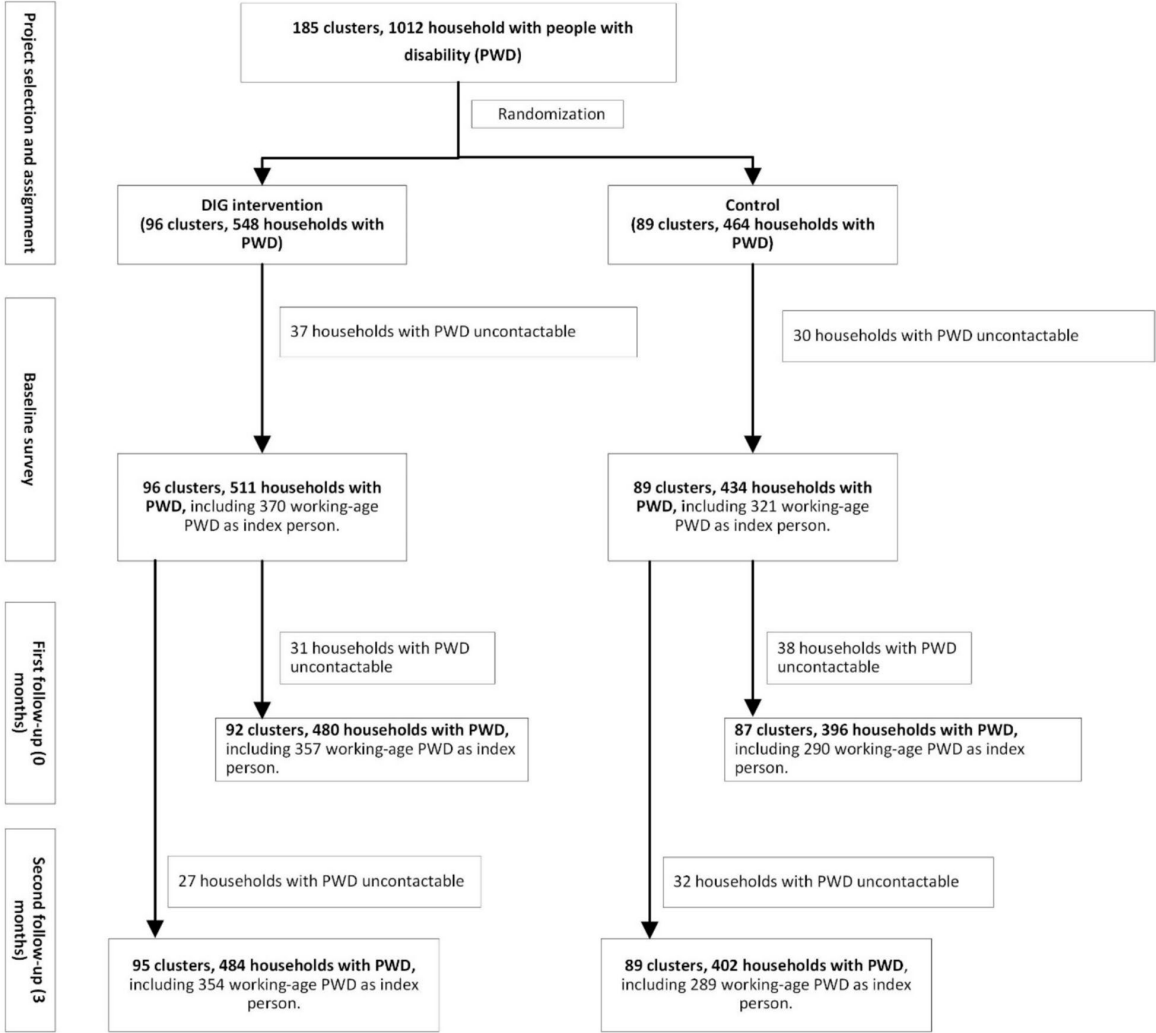
Trial profile. Flow diagram of cluster randomization, allocation to intervention and control arms, and follow-up status of participants. DIG, disability-inclusive graduation programme; PWD, people with disabilities

The programme team identified 5300 eligible households within the eight BRAC branches included in this study, with 320–420 households per branch (Fig. 1).

Randomization was conducted at the cluster level, stratified by BRAC office branch to ensure sufficient programmatic support while minimizing potential imbalance in confounding factors. Clusters were ranked based on the number of project participants who were people with disabilities, with geographical separation and cluster derivation also considered to minimize contamination.

Although initially aimed for a 1:1 allocation ratio within each branch, clusters were randomly assigned to the treatment group until reaching approximately 2700 households (the funder’s target). Ultimately, 96 clusters (2898 eligible households) were allocated to the DIG intervention group, and 89 clusters (2402 eligible households) to the control group (Fig. 1).

The randomization was performed after participant recruitment by an independent statistician using random number allocation in Stata (StataCorp. College Station, TX: StataCorp LLC) in October 2020, just before baseline data collection. While allocation concealment of participants or programme implementers was not possible due to the intervention’s nature, researchers responsible for recruitment and outcome data collection were masked to allocation status. All eligible households assumed the treatment status of their respective clusters.

### Procedures

The DIG programme was co-designed by BRAC, Humanity and Inclusion (HI), and National Union of Women with Disabilities of Uganda (NUWODU) through a nine-month collaborative process. The programme adopted a twin-track approach: providing personalized support to people with disabilities (including rehabilitation services and assistive aids) while mainstreaming disability inclusion across all four pillars of the graduation model.

Households in treatment clusters received the DIG programme between December 2020 and June 2022, with each batch receiving components for up to 18 months. The intervention packages included four graduation pillars: (1) Livelihoods: Technical training, asset transfer, and mentoring on income generation, with assets chosen based on local market opportunities and recipients’ preferences; (2) Social Protection: Six-month cash transfer (USD 18/month), emergency health fund subsidy, functional rehabilitation services, and linkage to pre-existing social entitlements and support services; (3) Financial Inclusion: Financial literacy training, ongoing coaching for financial management skills, and creation of Village Savings and Loans Associations (VSLAs); (4) Social Empowerment: Individual counselling, life-skills coaching, and formation of inclusive Village Poverty Reduction Committees (VPRCs). The intervention is further described elsewhere [15, 16].

Project staff (90 members) underwent comprehensive training in project methodology, disability inclusion, and participant targeting. Each BRAC branch office had a dedicated team for intervention delivery, with regular supervision by BRAC staff, HI, and NUWODU. During the COVID-19 pandemic, adaptations included virtual training to maintain service continuity.

Participant adherence was promoted through regular home visits, group meetings, and bi-weekly support from field staff, providing tailored guidance on life skills and livelihood management. The control group did not receive the DIG intervention but may have had access to other community programmes unrelated to DIG.

### Data collection

Data collection occurred at three time points: baseline (pre-intervention, November 2020), first follow-up (immediately following the 18-month DIG programme, June–July 2022), and second follow-up (16 months post-intervention, October–November 2023). At each point, trained data collectors from BIGD/IERC used SurveyCTO, an electronic data collection tool, to gather information from both control and intervention arms.

The process comprised two components: first, household-level information and data on all household members was collected, including socio-demographic factors, economic status, and disability status; second, for households with people with disabilities, one working-age person with a disability per household (“index person”) was randomly selected to respond to an in-depth questionnaire. This questionnaire included specific, structured questions aimed at capturing detailed quantitative measures on people with disabilities, including items about social participation.

Informed consent was obtained prior to each round of data collection. Interviewers provided participants with hard copies of information sheets and consent forms, reading contents aloud to ensure comprehension. For individuals with different impairments, consent procedures were adapted as necessary (e.g. using sign language for participants with profound hearing impairments). Written informed consent was obtained through participant-dated signature or thumbprint.

No methods changed after trial commencement.

### Outcomes

The primary outcome measure was the level of social participation of people with disabilities, assessed using the SINTEF Participation Survey [18]. The SINTEF Participation Survey used to assess social participation has been validated in low-income contexts, including other African countries [18]. The instrument was translated into local languages and pilot-tested to ensure cultural appropriateness and comprehensibility. The instrument focused on two key domains: household participation (consultation in household decisions, feeling involved in the family, and family involvement in conversations) and societal participation (attendance at social events, participation in traditional practices, involvement in community meetings, and voting). Each item was scored on a three-point scale: “no” (0), “sometimes” (1), or “yes” (2). The total score was calculated by summing all seven items (maximum raw score of 14), then converted to a scale of 0–100, with higher scores indicating greater participation.

We tracked social events that might lead to project discontinuation, including economic hardships and significant health issues. When necessary, targeted support was provided through economic diversification training, emergency health funds, and community sensitization efforts to maintain participant engagement.

### Statistical analysis

The sample size calculation was primarily based on the trial’s main outcome measure of household income [16]. With 370 index persons in the intervention group and 321 in the control group at baseline, our study was expected to detect an effect size of approximately 0.2 at 80% power, using a two-tailed test at the 5% significance level.

Statistical analyses followed the CONSORT guidelines using R software version 4.0.1. The analyses were prespecified and overseen by an independent Data Safety and Monitoring Board. The trial was registered with the Registry for International Development Impact Evaluations (RIDIE-STUDY-ID-626008898983a) and ISRCTN (ISRCTN-78592382).

Analyses included participants and households who completed at least one follow-up survey. Intervention effects were estimated using linear mixed-effects regression and reported as minimally-adjusted mean differences (MAMDs), fully-adjusted mean differences (FAMDs), and standardized mean differences (using Hedges’ method [19]), with corresponding 95% confidence intervals (CIs). The minimally-adjusted model included fixed effect for treatment status and random intercept for cluster and branch. Fully adjusted models additionally included variables found to be imbalanced by loss to follow-up or at baseline (*p* < 0.10). Restricted maximum likelihood was employed to fit the model.

To explore sex differences in intervention effects, we repeated the above analysis including the interaction term between treatment status and sex.

## Results

From Jan 2020 to March 2020, we screened 185 clusters across 164 villages, covering 1012 ultra-poor households with people with disabilities (representing 19.1% of the total ultra-poor 5300 households). The clusters were subsequently randomized, with 96 clusters to the intervention group (DIG) and 89 clusters to the control group (Fig. 1). At baseline, 511 households from 96 clusters in the intervention group and 434 households from 89 clusters in the control group were surveyed. Among them, 691 working-age people with disabilities were selected (370 in the intervention group and 321 in the control group) as indexed people with disabilities and completed the participants questionnaire. Of the indexed people with disabilities enrolled at baseline, 647 (93.6%) were successfully interviewed at the first follow-up: 357 (96.5%) from the intervention group and 290 (90.3%) from the control group); and 643 (93.1%) were successfully interviewed at the second follow-up: 354 (95.7%) from the intervention group and 289 (90.3%) from the control group).

Attrition rates differed between the intervention and control groups at the first follow-up (Additional file 1: Table S1; project participants lost to follow-up were younger; *p* = 0.06); as well as at the second follow-up (Additional file 1: Table S2; project participants lost to follow-up were younger (*p* = 0.03), more likely to be educated (*p* = 0.09), and had a higher per capital income (*p* = 0.03).

Table 1 presents a summary of the baseline characteristics of index person and their household level factors, all of which were largely balanced between the two groups (Additional file 1: Table S3 and Additional file 1: Table S4). The mean age was 35.71 years (SD 12.42) in the intervention group and 34.31 years (SD 11.97) in the control group, with similar proportions of females (53.5% intervention, 53.6% control). As noted in Sup Table 2, a lower proportion of participants in the intervention group were married or cohabiting (53.5%) compared to the control group (62.1%, *p* = 0.05). There were no obvious imbalances between the groups in terms of the social overall participation scores (72.45 (SD 25.88) in the intervention group vs 71.97 (SD 24.94) in the control group), as well as its subdomains: Domain 1: household participation (86.31 (SD 25.27) in the intervention group vs 85.57 (SD 25.13) in the control group); and Domain 2: societal participation (62.06 (SD 33.21) in the intervention group vs 61.77 (SD 30.86) in the control group) (Table 1).

**Fig. 2:**
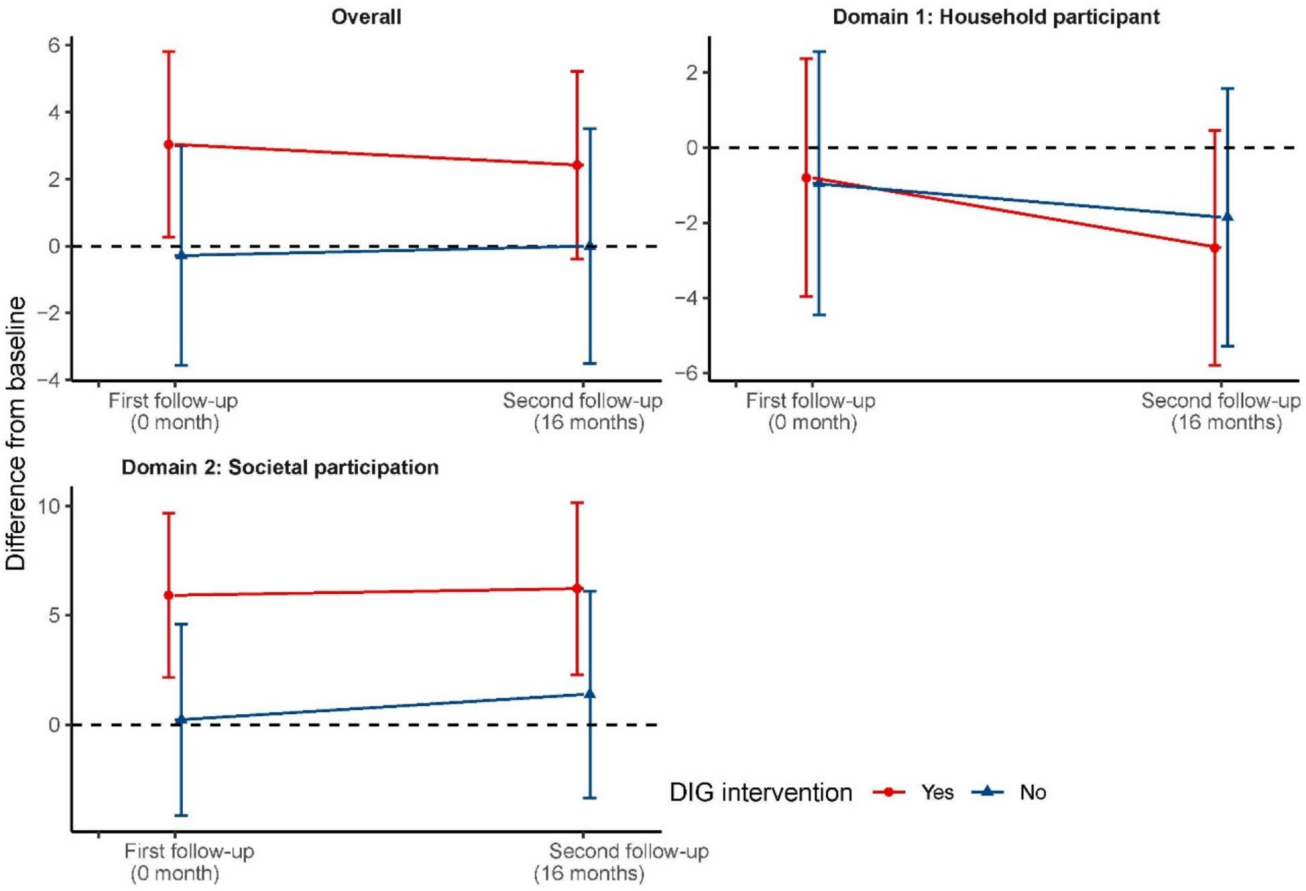
Outcomes across timepoints and comparison groups. Longitudinal changes in social participation outcomes by group (intervention vs control) and timepoint. Plotted points indicate mean change from baseline in overall social participation, household participation, and societal participation. Vertical lines represent 95% confidence intervals around the means. First follow-up was conducted immediately post-intervention, and second follow-up was conducted 16 months after the intervention ended

Figure 2 shows longitudinal changes in social participation scores from baseline, both overall and across two domains, across the two follow-ups. At immediately post-intervention (first follow-up), the DIG program shows potential positive effects on overall scores, with a notable increase in the intervention group compared to minimal change in the control group. This effect is particularly pronounced in Domain 2: Societal participation, where the intervention group demonstrates a substantial increase while the control group shows little change. For Domain 1: Household participation, both groups showed similar patterns of slight decrease by first follow-up. By 16 months post-intervention (second follow-up), the overall scores for the intervention group remain higher than baseline and the control group, though the difference appears to narrow. In Domain 1, both groups continue to show decreased scores from baseline, while in Domain 2, the intervention group maintains a higher increase compared to the control group, though the gap seems to reduce slightly compared to first follow-up.

Table 2 presents the outcomes across two follow-up waves (immediately post-intervention and 16 months post-intervention) for the DIG intervention and control groups. At the first follow-up, while the DIG intervention group showed an improvement in overall social participation (3.04 points) compared to a slight decrease in the control group (− 0.29 points), these changes were not statistically significant in either the minimally adjusted (MAMD 3.04, 95% CI − 1.98 to 8.04; *p* = 0.24; effect size 0.12, 95% CI − 0.08 to 0.31) or fully adjusted analyses (FAMD 3.14, 95% CI − 1.26 to 7.54; *p* = 0.17; effect size 0.14, 95% CI − 0.06 to 0.34). Domain 1 (Household participation) showed no difference between groups. Domain 2 (Societal participation) showed a larger improvement in the intervention group (5.92 points) compared to the control group (0.21 points), with the fully adjusted analysis approaching statistical significance (FAMD 5.84, 95% CI − 0.01 to 11.69; *p* = 0.05; effect size 0.20, 95% CI 0.00 to 0.41). At the second follow-up, the intervention group maintained a slight improvement in overall social participation (2.41 points) while the control group showed minimal change (− 0.01 points). The fully adjusted analysis indicated a positive but non-significant difference between groups (FAMD 2.56, 95% CI − 1.79 to 6.90; *p* = 0.25; effect size 0.11, 95% CI − 0.08 to 0.31). Domain 1 (Household participation) showed slight decreases in both groups, with no significant difference between them. Domain 2 (Societal participation) maintained a larger improvement in the intervention group (6.23 points) compared to the control group (1.38 points), though the difference was not statistically significant in the fully adjusted analysis (FAMD 4.85, 95% CI − 1.02 to 10.72; *p* = 0.11; effect size 0.17, 95% CI − 0.04 to 0.37).

**Table 2:** Effects of the disability-inclusive graduation programme across timepoints. Intervention effects were estimated using linear mixed-effects regression, reporting minimally adjusted mean differences (MAMDs) and fully adjusted mean differences (FAMDs) with 95% confidence intervals (CIs). The minimally adjusted model included treatment status (fixed effect) and cluster/branch (random intercepts). Fully adjusted models additionally controlled for imbalanced variables (*p* < 0.10): marital status and age of project participants for first follow-up, and these variables plus household per capita income for second follow-up

Outcomes	Outcome difference compared to baseline		Minimally adjusted analysis			Fully adjusted analysis		
	**DIG intervention**	**Control group**	**Mean difference (95% CI)**	***p* value**	**Effect size (95% CI)**	**Mean difference (95% CI)**	***p* value**	**Effect size (95% CI)**
**First follow-up (0 month after the intervention)**								
**Social participation**	3.04 (24.83)	− 0.29 (26.92)	3.04 [− 1.98, 8.04]	0.24	0.12 [− 0.08, 0.31]	3.14 [− 1.26, 7.54]	0.17	0.14 [− 0.06, 0.34]
Domain 1: Household participation	− 0.8 (28.4)	− 0.95 (28.81)	0.01 [− 4.92, 4.89]	1.00	0 [− 0.17, 0.17]	− 0.24 [− 4.49, 3.98]	0.91	− 0.01 [− 0.19, 0.17]
Domain 2: Societal participation	5.92 (33.65)	0.21 (36.08)	5.49 [− 1.59, 12.55]	0.13	0.16 [− 0.05, 0.36]	5.84 [− 0.01, 11.69]	0.05	0.20 [0, 0.41]
**Second follow-up (16 months after the intervention)**								
**Social participation**	2.41 (25.2)	− 0.01 (27.77)	2.25 [− 2.83, 7.28]	0.38	0.08 [− 0.11, 0.28]	2.56 [− 1.79, 6.90]	0.25	0.11 [− 0.08, 0.31]
Domain 1: Household participation	− 2.67 (28.04)	− 1.85 (27.1)	− 0.82 [− 5.45, 3.81]	0.73	− 0.03 [− 0.2, 0.14]	− 0.22 [− 4.12, 3.69]	0.91	− 0.01 [− 0.18, 0.16]
Domain 2: Societal participation	6.23 (35.37)	1.38 (37.44)	4.68 [− 2.56, 11.90]	0.20	0.13 [− 0.07, 0.33]	4.85 [− 1.02, 10.72]	0.11	0.17 [− 0.04, 0.37]

The analysis of the interaction between intervention and sex reveals no statistically significant differences in the effect of the intervention between males and females across both follow-ups and all outcome measures (Additional file 1: Table S5). The effects by sex and by follow-up were presented in Additional file 1: Table S6 and Additional file 1: Table S7. In the first follow-up, intervention group females showed a larger improvement in overall social participation scores (FAMD 5.92, 95% CI 0.36 to 11.45; *p* = 0.04; effect size 0.27, 95% CI 0.01 to 0.53) and Domain 2 societal participation scores (FAMD 8.22, 95% CI 1.16 to 15.25; *p* = 0.02; effect size 0.29, 95% CI 0.04 to 0.55), compared to control group females.

## Discussion

### Principle findings

This study contributes to the growing body of evidence on disability-inclusive graduation programmes by evaluating their effectiveness on social participation outcomes among ultra-poor households with disabilities in a low-income setting through a cluster randomized trial. While not the first of its kind, our study’s comprehensive design of the DIG intervention, which incorporates a twin-track approach to address the unique challenges faced by people with disabilities while fostering an inclusive environment, builds upon and extends previous poverty reduction initiatives. At 16-month follow-up, we found no significant long-term impact on overall social participation scores or on any of the two domains (household and societal participation). However, we observed some short-term effects at immediately post-intervention. At this time point, there was a positive effect on overall social participation scores favouring the intervention group, though not statistically significant in the fully adjusted analysis. Additionally, we observed a larger improvement in societal participation favouring the intervention group at immediately post-intervention, with the fully adjusted analysis approaching statistical significance. No differences were found in the domain of household participation at either follow-up. The analysis of the interaction between intervention and sex reveals no statistically significant differences in the effect of the intervention between males and females across both follow-up waves and all outcome measures.

### Comparisons and interpretations

The trends observed in overall social participation scores and societal participation at the 0-month follow-up suggest potential positive effects of the DIG programme, though these differences did not reach statistical significance in the fully adjusted analyses. This is consistent with our qualitative findings of the impact of the DIG programme, that persons with disabilities who participated in the DIG programme reported positive changes about the way they interacted and engaged in the community [20]. This short-term positive trend aligns with findings from several studies on disability-inclusive interventions. For instance, Butura et al. (2024) conducted a systematic review of the impact of CBR programmes and found that many interventions showed positive short-term effects on social participation and community engagement [13]. Our results are consistent with this trend, indicating that targeted, multifaceted interventions can yield rapid improvements in social inclusion for people with disabilities. Our findings also resonate with the work of Hunt et al. (2022), who found that disability-inclusive livelihood programmes could lead to quick gains in social participation and community involvement [11, 21]. Hunt et al. attributed these effects to the combination of economic empowerment and social support components, which is similar to the approach used in our DIG programme [21].

Our findings showed no change in household participation at any point in the study period, indicating that the DIG intervention did not effectively influence intra-household dynamics. This suggests that additional or different strategies may be needed to address household-level social participation.

The absence of significant long-term effects on social participation outcomes at the 16-month follow-up also warrants careful consideration. This finding suggests that while the DIG programme may have initial positive impacts, these effects may not be sustained over an extended period. Several factors may explain this lack of sustainability. First, the intensity and duration of the intervention (18 months) may have been insufficient to create lasting behavioural and social changes in deeply entrenched exclusionary practices. Second, without ongoing support mechanisms or “booster sessions” after the formal intervention ended, participants may have struggled to maintain new practices. Third, broader community and structural barriers likely persisted beyond the intervention period, undermining individual-level gains. Fourth, the cultural context in Northern Uganda, where traditional attitudes toward disability might be resistant to change, could have contributed to the regression toward pre-intervention patterns of participation. This finding aligns with some previous studies on poverty reduction interventions for people with disabilities. For instance, a systematic review of poverty reduction interventions for disabled individuals highlighted that while short-term improvements in economic stability and social inclusion are common, long-term sustainability often depends on ongoing policy support and targeted interventions that address the specific barriers faced by people with disabilities [22].

However, our results contrast with findings from certain CBR programmes. Two meta-analyses suggest that CBR may have positive long-term impacts on social participation for people with disabilities [23, 24]. The discrepancy between our findings and these more successful CBR programmes could relate to several key differences in implementation. First, many effective CBR programmes are characterized by longer intervention periods (often 3 + years compared to our 18 months) and more intensive community engagement components. Second, successful CBR programmes typically feature stronger integration with existing health and social services, creating sustainable support mechanisms. Third, some effective programmes incorporate explicit disability rights advocacy at community and governmental levels, addressing structural barriers more comprehensively than our DIG intervention. These differences suggest potential enhancements for future DIG designs. The discrepancy between our findings and previous studies could be attributed to differences in intervention design, intensity, duration, and primary outcome target. The lack of long-term impact in our study also diverges from some findings on general graduation programmes for ultra-poor populations. For example, Banerjee et al. (2015) found sustained positive effects of a multifaceted graduation approach on psychosocial outcomes in six countries, with impacts persisting 36 months post-intervention [25]. The difference in our results could be due to the unique challenges faced by people with disabilities, which may require more intensive or prolonged support to achieve lasting changes in social participation. It's important to note that our study’s focus on a disability-specific population and the comprehensive nature of the DIG intervention make direct comparisons with previous literature challenging. The complexity of addressing disability-related barriers to participation, combined with the multifaceted nature of poverty in this context, may contribute to the difficulty in sustaining long-term impacts. These findings highlight the need for further research into the factors that influence the sustainability of interventions aimed at improving social participation for people with disabilities in low-resource settings. Future studies might explore the potential benefits of extended intervention periods, more intensive follow-up support, or the integration of disability-specific components into existing community structures to promote lasting change.

Cultural context in Northern Uganda likely influenced our results, as traditional beliefs sometimes associate disability with supernatural causes, creating persistent stigma. The post-conflict environment, with underdeveloped infrastructure and rebuilding communities, presents additional challenges that may have affected intervention sustainability.

The analysis of the interaction between intervention and sex revealed no statistically significant differences in the effect of the intervention between males and females across both follow-up waves and all outcome measures. However, this result should be interpreted with caution. The absence of statistical significance does not necessarily mean there was no effect; it could be due to insufficient sample size to detect potentially meaningful differences, as it contrasts with some previous studies that have found gender differences in the effectiveness of poverty reduction and social inclusion interventions. For instance, a systematic review by Langer et al. (2018) on the effects of economic empowerment interventions for women in low- and middle-income countries found that impacts often varied by gender, with some interventions being more effective for women than men [26]. The sex-specific coefficients in Sup Table 6 and Sup Table 7 suggest a significantly stronger effect for females at the first follow-up, especially in the domain of societal participation. These findings highlight the complexity of gender dynamics in disability-inclusive interventions and suggest the need for larger sample sizes in future studies to more definitively assess potential gender differences. Additionally, qualitative research could provide valuable insights into the gender-specific experiences of participants and the factors influencing the sustainability of intervention effects for men and women with disabilities.

### Implications

The findings from this study on the DIG programme have significant implications for research, practice, and policy in disability-inclusive development. The short-term positive effects followed by diminishing impacts over time suggest a need to reconsider intervention design, duration, and sustainability strategies. The results imply that future interventions might benefit from extended durations, “booster” sessions, or ongoing support mechanisms to maintain initial gains. The differential effects across participation domains highlight the need for social participation-related multifaceted approaches tailored to specific areas of inclusion. For policymakers, these results suggest the need for creating enabling environments that support longer-term improvements beyond the intervention period. Policy enhancements could include strengthening Uganda’s disability legislation implementation through dedicated budgets, integrating disability services into existing systems, supporting disability organizations, and establishing formal linkages between graduation programmes and community structures. Methodologically, the study emphasizes the value of longitudinal research with extended follow-up periods. The varying effects across domains also point to the crucial role of contextual factors, aligning with Nilsen et al.’s (2019) emphasis on context-specific approaches [27].

### Strengths and limitations

This study demonstrates several strengths that enhance its contribution to the field of disability-inclusive development. Firstly, the use of a cluster randomized controlled trial design provides a robust methodological approach, allowing for causal inferences about the effectiveness of the DIG programme. Secondly, the study’s focus on ultra-poor households with disabilities in a low-income setting addresses a critical gap in the literature. The comprehensive nature of the DIG intervention, incorporating a twin-track approach, represents an innovative and holistic strategy for addressing the complex challenges faced by people with disabilities living in poverty. Another strength lies in the study’s longitudinal design with multiple follow-up points (immediately post-intervention and 16 months post-intervention), enabling the assessment of both short-term and longer-term impacts. The use of the SINTEF Participation survey as the primary outcome measure provides a multidimensional assessment of social participation, capturing various aspects of social inclusion.

This study also has several limitations. Firstly, the differential loss to follow-up between the intervention and control groups at both the 0-month and 16-month time points may have introduced bias, potentially affecting the validity of the results. Differential attrition likely occurred because control participants felt less engaged without intervention benefits, while intervention participants maintained regular contact with programme staff. Future studies could implement enhanced tracking procedures and modest participation incentives for better retention. Nevertheless, the similar conclusions from both MAMDs and FAMDs supported the robustness of our findings. Secondly, while the cluster randomization design helps mitigate contamination between groups, it may have reduced statistical power compared to individual randomization [28]. Alternative designs such as stepped-wedge approaches or increased sample sizes could address power limitations in future studies. The study’s focus on a specific geographical area in Uganda may limit the generalizability of findings to other contexts or populations. Thirdly, while the SINTEF Participation Survey used as the primary outcome measure is validated and multidimensional, it does not include domains on inclusion in employment, education, and disability-specific health care, which are crucial aspects of participation for people with disabilities. This omission may have limited the study’s ability to capture the full range of potential intervention effects. Fourthly, while the Washington Group Short Set (WG-SS) is widely used and offers a practical approach to identifying individuals with functional difficulties, it does not capture impairments related to mental health conditions comprehensively. Previous studies have highlighted this limitation, showing that the WG-SS may underestimate the prevalence of disability, particularly among those with mental health-related functional impairments [29, 30]. Fifthly, the reliance on self-reported measures for social participation may be subject to recall bias or social desirability effects, potentially influencing the accuracy of the outcomes [31]. Sixthly, the study’s inability to mask participants and program implementers to group allocation due to the nature of the intervention is another limitation, which could have introduced performance bias [32]. Future studies might use independent outcome assessors unaware of allocation status to minimize this bias. Lastly, while the study assessed outcomes up to 16 months post-intervention, even longer-term follow-up might have provided additional insights into the sustainability of effects.

These limitations should be considered when interpreting the study’s findings and could inform the design of future research in this field.

## Conclusions

The DIG programme showed trends toward improved social participation for ultra-poor people with disabilities in the short term, though these effects were not statistically significant. The lack of significant differences between groups, particularly in the longer term, highlights the complex nature of disability inclusion in resource-constrained settings. Future research and interventions should focus on strategies to maintain and build upon initial improvements, taking into account the multifaceted nature of participation and the specific contextual factors that influence long-term outcomes. This study contributes to the growing body of evidence on the efficacy of disability-inclusive interventions in LMICs and underscores the need for continued innovation and research in this critical area of global health and development.

## Supplementary Information

Additional file 1: Table S1 – Characteristics of index participants by loss to first follow-up. Table S2 – Characteristics of index participants by loss to second follow-up. Table S3 – Baseline characteristics of index participants with disabilities in intervention and control groups. Table S4 – Characteristics of project participants in intervention and control groups. Table S5 – Sex differences in intervention effects across timepoints. Table S6 – Intervention effects across timepoints among females with disabilities. Table S7 – Intervention effects across timepoints among males with disabilities.

## Data Availability

De-identified participant data will be made available upon reasonable request after the end of the project (anticipated May 2025). Interested researchers should contact the corresponding author to request access. Data will be shared in accordance with ethical approvals and institutional data sharing policies, and requests will be reviewed by the study team to ensure appropriate use.

## Abbreviations

BRAC: Bangladesh Rural Advancement Committee
CBR: Community-based rehabilitation
CI: Confidence interval
DIG: Disability-inclusive graduation
FAMD: Fully adjusted mean difference
FCDO: Foreign, Commonwealth and Development Office
HI: Humanity and inclusion
HIC: High-income country
ICF: International Classification of Functioning, Disability and Health
IERC: Independent Evaluation and Research Cell
ISRCTN: International Standard Randomised Controlled Trial Number
LMIC: Low- and middle-income country
LSHTM: London School of Hygiene & Tropical Medicine
MAMD: Minimally adjusted mean difference
NGO: Non-Governmental Organization
NIHR: National Institute for Health and Care Research
NUWODU: National Union of Women with Disabilities of Uganda
PENDA: Programme for Evidence to Inform Disability Action
RCT: Randomized controlled trial
RIDIE: Registry for International Development Impact Evaluations
SD: Standard deviation
SDG: Sustainable Development Goals
UN: United Nations
UNCRPD: United Nations Convention on the Rights of Persons with Disabilities
UNCST: Uganda National Council for Science and Technology
USD: United States Dollar
VPRC: Village Poverty Reduction Committee
VSLA: Village Savings and Loans Association
WG-SS: Washington Group Short Set
