# Supplementary material for: Disability-inclusive graduation programme intervention on social participation among ultra-poor people with disability in North Uganda: a cluster randomized trial
Source: BMC Med. 2025 Apr 30;23:253. doi: 10.1186/s12916-025-04100-3 (PMC12044952; doi:10.1186/s12916-025-04100-3)
Supplement: Supplementary file 1 — Additional file 1: Table S1 – Characteristics of index participants by loss to first follow-up. Table S2 – Characteristics of index participants by loss to second follow-up. Table S3 – Baseline characteristics of index participants with disabilities in intervention and control groups. Table S4 – Characteristics of project participants in intervention and control groups. Table S5 – Sex differences in intervention effects across timepoints. Table S6 – Intervention effects across timepoints among females with disabilities. Table S7 – Intervention effects across timepoints among males with disabilities. [file 12916_2025_4100_MOESM1_ESM.docx]

Additional file 1

**Table S1.** **Basic description by loss to first follow-up.** Data was reported as the mean (standard deviation) or number (percentage). P values were extracted from two-tailed test for continuous variables and two-tailed chi-square test for categorical variables.

|  | **Loss to first follow-up** | | **p value** |
| --- | --- | --- | --- |
|  | Yes (n = 118) | No (n = 573) |  |
| **Individual-level factors for index person** |  |  |  |
| **Age (years)** | 33.54 (12.91) | 35.37 (12.06) | 0.16 |
| **Sex (=female)** | 62 (52.5%) | 308 (53.8%) | 0.89 |
| **Level of education** |  |  |  |
| No education | 22 (18.6%) | 137 (23.9%) | 0.53 |
| Primary education | 78 (66.1%) | 356 (62.1%) |  |
| Secondary education | 15 (12.7%) | 72 (12.6%) |  |
| Specialized training/bachelor or above | 3 (2.5%) | 8 (1.4%) |  |
| **Marital status** |  |  |  |
| Never married | 39 (33.1%) | 194 (33.9%) | 0.58 |
| Married/cohabiting | 57 (48.3%) | 251 (43.8%) |  |
| Divorced/separated/widow | 22 (18.6%) | 128 (22.3%) |  |
| **Is household head (=yes)** | 54 (45.8%) | 260 (45.4%) | 1.00 |
| **Is project participant (=yes)** | 56 (47.5%) | 273 (47.6%) | 1.00 |
| **Social participation** | 70.71 (25.82) | 72.54 (25.36) | 0.48 |
| Domain 1: Household participation | 84.18 (26.32) | 86.33 (24.96) | 0.42 |
| Domain 2: Societal participation | 60.61 (31.35) | 62.19 (32.29) | 0.62 |
| **Individual-level factors for project Participants** |  |  |  |
| **Age (years)** | 39.77 (13.72) | 42.41 (14.23) | **0.06** |
| **Sex (=female)** | 88 (74.6%) | 406 (70.9%) | 0.48 |
| **Level of education** |  |  |  |
| No education | 22 (18.6%) | 116 (20.2%) | 0.43 |
| Primary education | 84 (71.2%) | 376 (65.6%) |  |
| Secondary education | 12 (10.2%) | 73 (12.7%) |  |
| Specialized training/bachelor or above |  | 8 (1.4%) |  |
| **Marital status** |  |  |  |
| Never married | 13 (11.0%) | 53 (9.3%) | 0.75 |
| Married/cohabiting | 69 (58.5%) | 326 (57.3%) |  |
| Divorced/separated/widow | 36 (30.5%) | 190 (33.4%) |  |
| **Is household head (=yes)** | 68 (57.6%) | 351 (61.3%) | 0.53 |
| **Household-level factors** |  |  |  |
| **Highest level of education** |  |  |  |
| No education | 1 (0.8%) | 7 (1.2%) | 0.41 |
| Primary education | 75 (63.6%) | 319 (55.7%) |  |
| Secondary education | 35 (29.7%) | 215 (37.5%) |  |
| Specialized training/bachelor or above | 7 (5.9%) | 32 (5.6%) |  |
| **Lives in poverty (=yes)** | 118 (100.0%) | 573 (100.0%) | - |
| **Household size** | 5.64 (2.01) | 5.82 (2.33) | 0.42 |
| Number of children in household | 0.72 (0.92) | 0.71 (0.96) | 0.89 |
| **Per capital income (dollars) per month** | 74.70 (78.82) | 66.68 (77.60) | 0.31 |

**Table S2.** **Basic description by loss to second follow-up.** Data was reported as the mean (standard deviation) or number (percentage). P values were extracted from two-tailed test for continuous variables and two-tailed chi-square test for categorical variables.

|  | **Loss to second follow-up** | | **p value** |
| --- | --- | --- | --- |
|  | Yes (136) | No (555) |  |
| **Individual-level factors for index person** |  |  |  |
| **Age (years)** | 34.32 (13.14) | 35.24 (11.99) | 0.46 |
| **Sex (=female)** | 73 (53.7%) | 297 (53.5%) | 1.00 |
| **Level of education** |  |  |  |
| No education | 35 (25.7%) | 124 (22.3%) | 0.26 |
| Primary education | 87 (64.0%) | 347 (62.5%) |  |
| Secondary education | 14 (10.3%) | 73 (13.2%) |  |
| Specialized training/bachelor or above |  | 11 (2.0%) |  |
| **Marital status** |  |  |  |
| Never married | 46 (33.8%) | 187 (33.7%) | 0.99 |
| Married/cohabiting | 61 (44.9%) | 247 (44.5%) |  |
| Divorced/separated/widow | 29 (21.3%) | 121 (21.8%) |  |
| **Is household head (=yes)** | 64 (47.1%) | 250 (45.0%) | 0.74 |
| **Is project participant (=yes)** | 73 (53.7%) | 256 (46.1%) | 0.14 |
| **Social participation** | 72.25 (26.79) | 72.22 (25.11) | 0.99 |
| Domain 1: Household participation | 83.82 (27.21) | 86.49 (24.66) | 0.30 |
| Domain 2: Societal participation | 63.57 (32.20) | 61.52 (32.11) | 0.51 |
| **Individual-level factors for project Participants** |  |  |  |
| **Age (years)** | 39.71 (13.14) | 42.51 (14.37) | 0.03 |
| **Sex (=female)** | 101 (74.3%) | 393 (70.8%) | 0.49 |
| **Level of education** |  |  |  |
| No education | 35 (25.7%) | 103 (18.6%) | 0.09 |
| Primary education | 89 (65.4%) | 371 (66.8%) |  |
| Secondary education | 12 (8.8%) | 73 (13.2%) |  |
| Specialized training/bachelor or above |  | 8 (1.4%) |  |
| **Marital status** |  |  |  |
| Never married | 13 (9.6%) | 53 (9.6%) | 0.73 |
| Married/cohabiting | 82 (60.3%) | 313 (56.8%) |  |
| Divorced/separated/widow | 41 (30.1%) | 185 (33.6%) |  |
| **Is household head (=yes)** | 75 (55.1%) | 344 (62.0%) | 0.17 |
| **Household-level factors** |  |  |  |
| **Highest level of education** |  |  |  |
| No education | 1 (0.7%) | 7 (1.3%) | 0.19 |
| Primary education | 85 (62.5%) | 309 (55.7%) |  |
| Secondary education | 47 (34.6%) | 203 (36.6%) |  |
| Specialized training/bachelor or above | 3 (2.2%) | 36 (6.5%) |  |
| **Lives in poverty (=yes)** | 136 (100.0%) | 555 (100.0%) | - |
| **Household size** | 5.79 (2.05) | 5.79 (2.33) | 1.00 |
| Number of children in household | 0.65 (0.84) | 0.72 (0.98) | 0.41 |
| **Per capital income (dollars) per month** | 82.70 (89.84) | 64.46 (74.22) | 0.03 |

**Table S3.** **Baseline characteristics of index people with disabilities in the DIG intervention and control groups.** Data was reported as the mean (standard deviation) or number (percentage). P values were extracted from two-tailed test for continuous variables and two-tailed chi-square test for categorical variables.

|  | **DIG Intervention group**  **(n = 370)** | **Control group**  **(n = 321)** | **p value** |
| --- | --- | --- | --- |
| **Individual-level factors** |  |  |  |
| **Age (years)** | 35.71 (12.42) | 34.31 (11.97) | 0.14 |
| **Sex (=female)** | 198 (53.5%) | 172 (53.6%) | 1.00 |
| **Level of education** |  |  |  |
| No education | 82 (22.2%) | 77 (24.0%) | 0.19 |
| Primary education | 226 (61.1%) | 208 (64.8%) |  |
| Secondary education | 56 (15.1%) | 31 (9.7%) |  |
| Specialized training/bachelor or above | 6 (1.6%) | 5 (1.6%) |  |
| **Marital status** |  |  |  |
| Never married | 123 (33.2%) | 110 (34.3%) | 0.13 |
| Married/cohabiting | 156 (42.2%) | 152 (47.4%) |  |
| Divorced/separated/widow | 91 (24.6%) | 59 (18.4%) |  |
| **Is household head (=yes)** | 173 (46.8%) | 141 (43.9%) | 0.50 |
| **Is project participant (=yes)** | 176 (47.6%) | 153 (47.7%) | 1.00 |
| **Social participation** | 72.45 (25.88) | 71.97 (24.94) | 0.80 |
| Domain 1: Household participation | 86.31 (25.27) | 85.57 (25.13) | 0.70 |
| Domain 2: Societal participation | 62.06 (33.21) | 61.77 (30.86) | 0.90 |
| **Household-level factors** |  |  |  |
| **Highest level of education** |  |  |  |
| No education | 3 (0.8%) | 5 (1.6%) | 0.64 |
| Primary education | 206 (55.7%) | 188 (58.6%) |  |
| Secondary education | 140 (37.8%) | 110 (34.3%) |  |
| Specialized training/bachelor or above | 21 (5.7%) | 18 (5.6%) |  |
| **Lives in poverty (=yes)** | 370 (100.0%) | 321 (100.0%) | - |
| **Household size** | 5.78 (2.18) | 5.79 (2.38) | 0.93 |
| Number of children in household | 0.67 (0.91) | 0.75 (1.00) | 0.29 |
| **Per capital income (dollars) per month** | 69.33 (80.11) | 66.58 (75.16) | 0.64 |

**Table S4.** **Basic description of project participants.** Data was reported as the mean (standard deviation) or number (percentage). P values were extracted from two-tailed test for continuous variables and two-tailed chi-square test for categorical variables.

|  | **DIG Intervention group**  **(n = 370)** | **Control group**  **(n = 321)** | **p value** |
| --- | --- | --- | --- |
| **Age (years)** | 42.26 (14.32) | 41.61 (14.00) | 0.54 |
| **Sex (=female)** | 270 (73.0%) | 224 (69.8%) | 0.40 |
| **Level of education** |  |  |  |
| No education | 70 (18.9%) | 68 (21.2%) | 0.84 |
| Primary education | 250 (67.6%) | 210 (65.4%) |  |
| Secondary education | 45 (12.2%) | 40 (12.5%) |  |
| Specialized training/bachelor or above | 5 (1.4%) | 3 (0.9%) |  |
| **Marital status** |  |  |  |
| Never married | 35 (9.5%) | 31 (9.7%) | **0.05** |
| Married/cohabiting | 197 (53.5%) | 198 (62.1%) |  |
| Divorced/separated/widow | 136 (37.0%) | 90 (28.2%) |  |
| **Is household head (=yes)** | 231 (62.4%) | 188 (58.6%) | 0.34 |

**Table S5. Sex difference on the effects of the Disability-inclusive graduation programme across timepoints**, **with male as the reference**. Intervention effects were estimated using linear mixed-effects regression, reporting minimally-adjusted mean differences (MAMDs) and fully-adjusted mean differences (FAMDs) with 95% confidence intervals (CIs). The minimally-adjusted model included treatment status (fixed effect) and cluster/branch (random intercepts). Fully adjusted models additionally controlled for imbalanced variables (p<0.10): marital status and age of project participants for first follow-up; and these variables plus household per capita income for second follow-up. Sex differences in intervention effects were estimated by including the interaction term between treatment status and sex.

| **Outcomes** | **Outcome difference compared to baseline** | | | | **Minimally adjusted analysis** | | | **Fully adjusted analysis** | | |
| --- | --- | --- | --- | --- | --- | --- | --- | --- | --- | --- |
|  | **Males** | | **Females** | |  |  |  |  |  |  |
|  | **DIG intervention** | **Control group** | **DIG intervention** | **Control group** | **Mean difference (95% CI)** | **p value** | **Effect size (95% CI)** | **Mean difference (95% CI)** | **p value** | **Effect size (95% CI)** |
| **First follow-up (0 month after the intervention)** |  |  |  |  |  |  |  |  |  |  |
| **Social participation** | 0.11 (25.44) | -1.1 (24.89) | 5.43 (24.13) | 0.38 (28.55) | 3.12 [-5.27, 11.52] | 0.47 | -0.05 [-0.39, 0.28] | 4.76 [-2.41, 11.93] | 0.20 | 0.21 [-0.11, 0.54] |
| Domain 1: Household participation | -3.45 (31.45) | -1.84 (27.11) | 1.36 (25.53) | -0.23 (30.21) | 3.19 [-6.23, 12.61] | 0.51 | -0.03 [-0.36, 0.31] | 7.02 [-0.86, 14.89] | 0.08 | 0.29 [-0.04, 0.62] |
| Domain 2: Societal participation | 2.79 (32.84) | -0.55 (32.94) | 8.48 (34.17) | 0.83 (38.57) | 2.88 [-8.33, 14.10] | 0.61 | -0.05 [-0.39, 0.28] | 3.67 [-5.50, 12.82] | 0.43 | 0.13 [-0.19, 0.45] |
| **Second follow-up (16 months after the intervention)** |  |  |  |  |  |  |  |  |  |  |
| **Social participation** | 0.08 (27.56) | -2.29 (25.36) | 4.24 (23.11) | 2.02 (29.69) | -1.36 [-10.21, 7.49] | 0.76 | -0.05 [-0.39, 0.28] | -0.23 [-7.70, 7.30] | 0.95 | -0.01 [-0.35, 0.33] |
| Domain 1: Household participation | -3.41 (31.25) | -2.92 (27) | -2.1 (25.31) | -0.9 (27.27) | -0.71 [-10.01, 8.59] | 0.88 | -0.03 [-0.36, 0.31] | 1.57 [-6.27, 9.41] | 0.70 | 0.07 [-0.27, 0.41] |
| Domain 2: Societal participation | 2.70 (35.46) | -1.82 (33.94) | 8.99 (35.16) | 4.21 (40.21) | -1.93 [-14.06, 10.21] | 0.75 | -0.05 [-0.39, 0.28] | -0.80 [-10.24, 8.67] | 0.87 | -0.03 [-0.36, 0.31] |

**Table S6. Effects of the Disability-inclusive graduation programme across timepoints, among females with disabilities**. Intervention effects were estimated using linear mixed-effects regression, reporting minimally-adjusted mean differences (MAMDs) and fully-adjusted mean differences (FAMDs) with 95% confidence intervals (CIs). The minimally-adjusted model included treatment status (fixed effect) and cluster/branch (random intercepts). Fully adjusted models additionally controlled for imbalanced variables (p<0.10): marital status and age of project participants for first follow-up; and these variables plus household per capita income for second follow-up.

| **Outcomes** | **Outcome difference compared to baseline** | | **Minimally adjusted analysis** | | | **Fully adjusted analysis** | | |
| --- | --- | --- | --- | --- | --- | --- | --- | --- |
|  | **DIG intervention** | **Control group** | **Mean difference or odds ratio (95% CI)*** | **p value** | **Effect size (95% CI)** | **Mean difference or odds ratio (95% CI)*** | **p value** | **Effect size (95% CI)** |
| **First follow-up (0 month after the intervention)** |  |  |  |  |  |  |  |  |
| **Social participation** | 5.43 (24.13) | 0.38 (28.55) | 4.78 [-1.40, 10.88] | 0.13 | 0.18 [-0.05, 0.42] | 5.92 [0.36, 11.45] | **0.04** | 0.27 [0.01, 0.53] |
| Domain 1: Household participation | 1.36 (25.53) | -0.23 (30.21) | 1.53 [-5.16, 8.21] | 0.66 | 0.05 [-0.19, 0.3] | 2.98 [-2.99, 8.93] | 0.33 | 0.13 [-0.13, 0.38] |
| Domain 2: Societal participation | 8.48 (34.17) | 0.83 (38.57) | 6.97 [-1.78, 15.62] | 0.12 | 0.19 [-0.05, 0.43] | 8.22 [1.16, 15.25] | 0.02 | 0.29 [0.04, 0.55] |
| **Second follow-up (16 months after the intervention)** |  |  |  |  |  |  |  |  |
| **Social participation** | 4.24 (23.11) | 2.02 (29.69) | 1.54 [-5.09, 7.96] | 0.64 | 0.06 [-0.19, 0.31] | 1.80 [-4.25, 7.80] | 0.56 | 0.08 [-0.19, 0.35] |
| Domain 1: Household participation | -2.1 (25.31) | -0.9 (27.27) | -1.19 [-7.14, 4.76] | 0.69 | -0.05 [-0.27, 0.18] | 0.22 [-5.08, 5.51] | 0.94 | 0.01 [-0.23, 0.25] |
| Domain 2: Societal participation | 8.99 (35.16) | 4.21 (40.21) | 3.65 [-6.12, 13.26] | 0.46 | 0.1 [-0.16, 0.36] | 3.65 [-4.36, 11.63] | 0.37 | 0.12 [-0.15, 0.4] |

**Table S7. Effects of the Disability-inclusive graduation programme across timepoints, among males with disabilities**. Intervention effects were estimated using linear mixed-effects regression, reporting minimally-adjusted mean differences (MAMDs) and fully-adjusted mean differences (FAMDs) with 95% confidence intervals (CIs). The minimally-adjusted model included treatment status (fixed effect) and cluster/branch (random intercepts). Fully adjusted models additionally controlled for imbalanced variables (p<0.10): marital status and age of project participants for first follow-up; and these variables plus household per capita income for second follow-up.

| **Outcomes** | **Outcome difference compared to baseline** | | **Minimally adjusted analysis** | | | **Fully adjusted analysis** | | |
| --- | --- | --- | --- | --- | --- | --- | --- | --- |
|  | **DIG intervention** | **Control group** | **Mean difference or odds ratio (95% CI)*** | **p value** | **Effect size (95% CI)** | **Mean difference or odds ratio (95% CI)*** | **p value** | **Effect size (95% CI)** |
| **First follow-up (0 month after the intervention)** |  |  |  |  |  |  |  |  |
| **Social participation** | 0.11 (25.44) | -1.1 (24.89) | 1.73 [-4.93, 8.45] | 0.61 | 0.07 [-0.2, 0.33] | 1.15 [-4.78, 7.13] | 0.71 | 0.05 [-0.22, 0.32] |
| Domain 1: Household participation | -3.45 (31.45) | -1.84 (27.11) | -1.64 [-8.85, 5.62] | 0.66 | -0.06 [-0.3, 0.19] | -3.27 [-9.57, 3.04] | 0.31 | -0.13 [-0.38, 0.12] |
| Domain 2: Societal participation | 2.79 (32.84) | -0.55 (32.94) | 4.95 [-4.17, 14.18] | 0.29 | 0.15 [-0.13, 0.43] | 4.47 [-3.50, 12.50] | 0.28 | 0.16 [-0.13, 0.44] |
| **Second follow-up (16 months after the intervention)** |  |  |  |  |  |  |  |  |
| **Social participation** | 0.08 (27.56) | -2.29 (25.36) | 3.65 [-3.82, 11.28] | 0.34 | 0.14 [-0.15, 0.42] | 3.14 [-3.20, 9.58] | 0.34 | 0.14 [-0.15, 0.42] |
| Domain 1: Household participation | -3.41 (31.25) | -2.92 (27) | 0.14 [-8.15, 8.48] | 0.97 | 0.01 [-0.28, 0.29] | -0.71 [-7.40, 6.06] | 0.84 | -0.03 [-0.3, 0.24] |
| Domain 2: Societal participation | 2.7 (35.46) | -1.82 (33.94) | 5.95 [-3.54, 15.69] | 0.22 | 0.17 [-0.1, 0.45] | 5.92 [-1.94, 13.89] | 0.15 | 0.21 [-0.08, 0.5] |
